# Comparison of Arctic Front Advance Pro and POLARx cryoballoons for ablation therapy of atrial fibrillation: an intraprocedural analysis

**DOI:** 10.1007/s00392-024-02398-2

**Published:** 2024-02-15

**Authors:** Vincent Knappe, Caroline Lahrmann, Maximilian Funken, Andreas Zietzer, Christopher Gestrich, Georg Nickenig, Jan W. Schrickel, Thomas Beiert

**Affiliations:** Heart Center Bonn, Department of Internal Medicine II, University Hospital Bonn, Venusberg-Campus 1, 53127 Bonn, Germany

**Keywords:** Atrial fibrillation, Cryoballoon, Cryoablation, Pulmonary vein isolation

## Abstract

**Introduction:**

Cryoballoon (CB) ablation has become a popular method for pulmonary vein isolation (PVI) in atrial fibrillation (AF) treatment. This study aimed to compare the intraprocedural ablation characteristics of two cryoballoons, Arctic Front Advance Pro™ (AFA-Pro, Medtronic) and POLARx™ (Boston Scientific).

**Methods and results:**

In this retrospective single-center study, 230 symptomatic paroxysmal or persistent AF patients underwent CB ablation with either AFA-Pro or POLARx. Propensity-score matching resulted in two cohorts of 114 patients each. Baseline and procedural characteristics were comparable between both CBs. POLARx achieved lower minimal temperatures (e.g., left superior pulmonary vein, LSPV: AFA-Pro − 49.0 °C vs. POLARx − 59.5 °C) and lower temperatures at time-to-isolation (TTI). Additionally, POLARx reached lower temperatures faster, as evidenced by lower temperatures after 40 and 60 s, and a larger mean temperature change between 20 and 40 s. POLARx also had a greater area under the curve below 0 °C and a longer thawing phase. Both CBs achieved comparable high rates of final PV-isolation.

TTI, minimal esophagus temperature, and first-pass isolation rates were similar between groups. Periprocedural complications, including phrenic nerve injuries, were comparable. Troponin levels in the left atrium were elevated with both systems. Values and change in troponin were numerically higher in the POLARx group (delta troponin: AFA-Pro 36.3 (26.4, 125.4) ng/L vs. POLARx 104.9 (49.5, 122.2) ng/L),* p* = 0.077).

**Conclusion:**

AFA-Pro and POLARx are both highly effective and safe CB systems for PVI. POLARx exhibited significant faster and lower freezing characteristics, and numerically higher troponin levels might indicate greater myocardial injury. However, these differences did not translate into improved performance, procedural efficiency, or safety.

**Graphical abstract:**

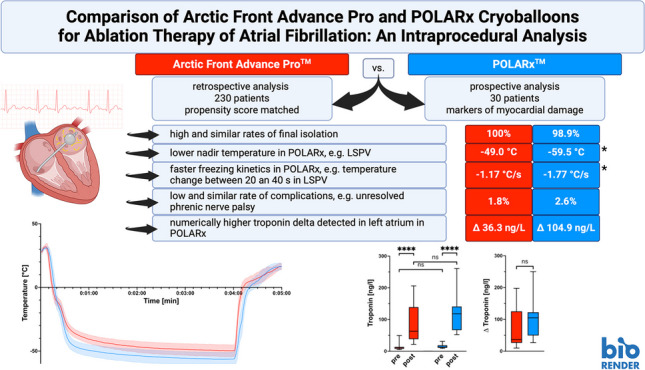

**Supplementary Information:**

The online version contains supplementary material available at 10.1007/s00392-024-02398-2.

## Introduction

The development of effective therapeutic strategies in atrial fibrillation (AF) is of utmost importance due to increasing incidence and prevalence of the disease [1]. In recent years, ablation therapy has emerged as a promising treatment option [2, 3], especially when administered early in the course of the disease. Notably, landmark studies like EAST-AFNET 4 [4], STOP-AF [5], and EARLY-AF [6] have underscored the efficacy of an early ablation therapy.

Cryoballoon (CB) ablation has gained increasing attention due to its simplicity, safety, and efficacy in pulmonary vein isolation (PVI). The Arctic Front Advance™ (AFA) system, developed by Medtronic (Medtronic, USA), has been the most established cryoballoon system, with the Arctic Front Advance Pro™ (AFA-Pro) representing the latest fourth-generation iteration [7]. However, the landscape of CB ablation has evolved with the introduction of the POLARx™ CB system by Boston (Boston Scientific, USA) [8–10]. Initial studies have reported lower minimal temperatures and higher rates of time-to-isolation (TTI) recordings with POLARx [8, 11]. Nevertheless, a comprehensive understanding of the freezing characteristics of these two CBs and their implications on tissue damage during ablation remains elusive, with previous studies being limited to small patient cohorts. Furthermore, it remains crucial to investigate whether differences in freezing profiles between the two CBs translate into altered tissue damage during ablation, potentially leading to differences in acute and especially long-term ablation success and efficacy. Additionally, a comparative examination of both CB systems is also necessary with regard to potential complications such as lower esophageal temperatures or increased phrenic nerve paresis. Hence, the aim of this study is to provide a comprehensive and detailed comparison of the freezing characteristics of AFA-Pro and POLARx with particular focus on intraprocedural ablation characteristics, acute ablation success, efficacy, and safety. Troponin measurements taken from the left atrium (LA) before and after ablation will serve as a quantifiable marker to assess the potential impact of altered freezing properties on myocardial tissue damage during the ablation procedure.

## Material and methods

### Study design and population

This study was conducted at the tertiary care university hospital in Bonn, Germany. The retrospective, observational patient cohort was recruited during the period from March 2020 to June 2022. After screening of 519 patients scheduled for an AF ablation procedure, a total of 230 patients with symptomatic paroxysmal or persistent AF were included in the study. Exclusion criteria were patients with long-standing persistent AF, previous surgical or catheter-based ablation, presence of left atrial (LA) thrombus in transesophageal echocardiography prior to the procedure, severe valvular heart disease, or contraindications to postprocedural oral anticoagulation. All procedures were performed by experienced electrophysiologists with a high level of expertise in CB procedures.

In addition to the main study population described above, a second study cohort was included specifically for troponin measurements from the left atrium before and after ablation. This subpopulation (troponin cohort) consisted of 30 patients (173 screened) who were prospectively enrolled and received PVI using either AFA-Pro (14 patients) or POLARx (16 patients). The inclusion and exclusion criteria for this cohort were identical to those outlined for the primary study population.

The study complies with the Declaration of Helsinki and was approved by the local ethics committee (321/21 and 460/21).

### Preprocedural management

Before undergoing PVI, a comprehensive transthoracic and transesophageal echocardiography was performed in all patients. Two-dimensional (2D) echocardiography was used to determine left ventricular ejection fraction (LVEF), left atrial (LA) volume, left ventricular (LV) end-diastolic volume, systolic pulmonary artery pressure (sPAP), mitral regurgitation (MR), and left atrial appendage (LAA) velocity. The presence of any cardiac thrombus was ruled out in all patients.

Antiarrhythmic drugs (AAD) were continued until the scheduled ablation date. Subsequent to the procedure, these medications were discontinued, except for beta-blockers. In patients receiving new oral anticoagulants, the morning dose on the day of the procedure was withheld. For patients taking vitamin K antagonists, the procedure was conducted when their international normalized ratio (INR) values were therapeutically maintained between 2 and 3.

### Cryoballoon ablation

Ablation was performed in patients under procedural sedation and analgesia with midazolam and pethidine. A temperature probe was routinely placed in the esophagus to monitor temperature changes during the freezing cycles. Access for the PVI was obtained through the right femoral vein. A diagnostic multipolar catheter was inserted and positioned in the coronary sinus (CS) via central venous access. Single transseptal puncture was performed using fluoroscopic guidance, employing the Brockenbrough technique, and the steerable 15 F sheath (Flexcath™, Medtronic, USA, or Polarsheath™, Boston Scientific, USA) was introduced under fluoroscopic guidance into the LA. Subsequently, the AFA-Pro CB or POLARx CB were inserted and carefully positioned proximal to the PV ostium. After inflation, the devices were advanced and pushed to the ostium to occlude it completely. Optimal and effective occlusion of the PVs was verified by contrast application. To maximize TTI recordings, the spiral mapping catheter (Achieve™, Medtronic, USA, or Polarmap™, Boston Scientific, USA) was retracted as close as possible to the PV ostium without compromising PV occlusion.

The freezing process was typically set for 240 s. Adjustments were possible based on TTI, temperature changes, minimal temperature, esophageal temperature, and others. Criteria for premature termination were a freezing temperature of − 70 °C for POLARx (mandatory technical parameter of CB system), an esophageal temperature below 16 °C, or attenuation of phrenic nerve capture.

While performing the ablation procedure on the right PVs, the function of the phrenic nerve was observed through continuous phrenic nerve pacing using a diagnostic catheter positioned in the superior vena cava. Diaphragmatic excursion was assessed by continuous abdomen palpation by the investigator and intermittent fluoroscopy, with immediate interruption of ablation on detection of attenuated diaphragmatic movements. Additionally, in the POLARx group, the newly introduced diaphragm movement sensor (DMS) from Boston was used to monitor the function of the phrenic nerve. To verify ablation success, the spiral mapping catheter was re-advanced in the targeted PVs. Elimination of all PV potentials was defined as entrance block. Exit block was evaluated by electrical stimulation within each PV.

Throughout the procedure, intravenous heparin was administered to achieve an activated clotting time of > 300 s after transseptal puncture.

### Postprocedural care

At the end of the procedure, closure of the vascular access site was achieved by manual compression, a figure-of-eight suture, and a pressure bandage for 6 h. Unless bleeding complications occurred, the anticoagulation with new oral anticoagulants and vitamin K antagonists was re-initiated 8 h after the ablation. Anticoagulation was administered for a minimum of 3 months post-procedure and then continued based on the individual CHA_2_DS_2_-VASc score. To rule out pericardial effusion, echocardiographic examinations were performed immediately after the procedure, on arrival to the ward, and the following day. All patients were advised to take a PPI for 4 weeks to prevent atrio-oesophageal fistula. Previously prescribed antiarrhythmic drugs were discontinued individually for each patient.

### Laboratory analysis

As part of the preprocedural preparation, peripheral blood was drawn from all patients, and blood count, coagulation parameters, renal function parameters, thyroid levels, and, in some cases, inflammation levels were determined.

In our second study cohort, we collected blood samples from the left atrium for troponin measurements: blood was collected immediately after transseptal puncture and at the end of the procedure upon verification of ablation success. Troponin T levels were measured using a hs-cTnT assay (Elecsys, Roche Diagnostics, Germany) with a 99th percentile upper reference limit of 14 ng/L.

### Statistical analysis

We performed propensity score matching of patients treated with POLARx or AFA-Pro in a 1:1 ratio with a match tolerance of 0.1. Multivariable logistic regression was used to calculate propensity scores for each patient based on sex, age, paroxysmal AF, CHA_2_DS_2_-VASc, hypertension, diabetes, LVEF, and LA volume as covariates.

All continuous variables were tested for normality using the Shapiro–Wilk test. Normally distributed variables are presented as mean with standard deviation. The unpaired two-tailed student’s *t*-test was performed for analysis. For non-normally distributed variables, a median with interquartile range (25th–75th percentile) was reported, and a Mann–Whitney U test was conducted. Categorical variables are expressed as frequencies and percentages and were analyzed using Pearson’s chi-square test and Fisher’s exact test, respectively.

The statistics performed are based on a two-sided significance level of 0.05. SPSS software version 27.0 (IBM Corp., USA) was used for statistical analyses. Graphs were created using GraphPad Prism software version 9.5.1 (GraphPad Software, Inc., USA).

## Results

### Patient characteristics

A total of 230 patients who underwent cryoballoon ablation using either AFA-Pro or POLARx were included in this study. Propensity score matching with a 1:1 ratio (pair-matching) was performed, creating two cohorts of 114 patients each. The average age of the patients was 67.6 ± 10.6 years, with 62.3% being male. The median CHA_2_DS_2_-VASc score was 2.8 ± 1.56. Except for a history of persistent AF, both cohorts did not exhibit any significant differences in baseline characteristics (detailed characteristics are provided in Table 1). The medication was comparable between the AFA-Pro and POLARx groups (Supplementary Table S1). Pre-procedural laboratory parameters, including kidney and thyroid function, blood count, and inflammation markers, did not show significant differences (Supplementary Table S2).

**Table 1:** Baseline characteristics of study population

	All patients (*n* = 228)	AFA-Pro (*n* = 114)	POLARx (*n* = 114)	*P* value
Age [years]	67.6 ± 10.6	68.0 ± 11.1	67.2 ± 10.1	0.587
Male sex (%)	142 (62.3)	71 (62.3)	71 (62.3)	1.000
Persistent AF (%)	102 (44.7)	59 (51.8)	43 (37.7)	0.033*
CHA_2_DS_2_-VASc score	2.8 ± 1.6	2.9 ± 1.5	2.7 ± 1.7	0.406
HASBLED score	1.6 ± 0.9	1.6 ± 0.8	1.6 ± 0.9	1.000
Hypertension (%)	180 (78.9)	88 (77.2)	92 (80.7)	0.516
Dyslipidemia (%)	78 (34.2)	44 (38.6)	34 (29.8)	0.163
BMI [kg/m^2^]	27.5 ± 4.1	27.7 ± 4.5	27.2 ± 3.8	0.447
Obesity (%)	63 (27.8)	33 (28.9)	30 (26.5)	0.687
Diabetes (%)	36 (15.8)	21 (18.4)	15 (13.2)	0.276
Coronary artery disease (%)	57 (25.0)	28 (24.6)	29 (25.4)	0.878
Congestive heart failure (%)	23 (10.1)	12 (10.5)	11 (9.6)	0.826
Peripheral artery disease (%)	15 (6.6)	11 (9.6)	4 (3.5)	0.061
Carotid artery disease (%)	22 (9.6)	12 (10.5)	10 (8.8)	0.654
Previous stroke (%)	15 (6.6)	6 (5.3)	9 (7.9)	0.423
Previous TIA (%)	8 (3.5)	4 (3.5)	4 (3.5)	1.000
COPD (%)	14 (6.1)	9 (7.9)	5 (4.4)	0.270
Sleep apnea (%)	21 (9.2)	11 (9.6)	10 (8.8)	0.819

Values are *n* (%), mean ± standard deviation, or median (25th–75th percentile)

*AF*, atrial fibrillation; *BMI*, body mass index. Obesity was defined as BMI ≥ 30 kg/m^2^. *TIA*, transient ischaemic attack; *COPD*, chronic obstructive pulmonary disease

Echocardiographic characteristics of the study population were assessed through pre-procedural examinations, as outlined in Supplementary Table S3. The left ventricular ejection fraction was comparable between the groups (AFA-Pro: 57.8% (55.1, 63.6), POLARx: 58.1% (55.7, 63.5), *p* = 0.723). Furthermore, no significant differences were observed in left atrial volume between the groups, with values of 60.0 mL (45.8, 85.9) for AFA-Pro and 57.7 mL (43.7, 81.4) for POLARx (*p* = 0.534).

### Procedural characteristics

Among the 228 patients included in this study, 142 individuals (62.3%) presented with sinus rhythm as the preprocedural rhythm (Table 2). The median duration of the ablation procedure was comparable between the two CBs with 70.0 min (53.0, 85.0) for AFA-Pro and 70.0 min (55.0, 90.0) for POLARx (*p* = 0.238). There was no difference in the average amount of contrast medium used during the procedure (AFA-Pro 18.0 ± 5.0 mL vs. POLARx 18.4 ± 6.7 mL, *p* = 0.605). Regarding fluoroscopy time, the median duration was 10.0 min (8.2, 14.2) for AFA-Pro and 11.0 min (8.4, 14.6) for POLARx (*p* = 0.414).

**Table 2:** Procedural characteristics and adverse events

	All patients (*n* = 228)	AFA-Pro (*n* = 114)	POLARx (*n* = 114)	*P* value
Preprocedural rhythm (%)				0.751
Sinus rhythm	142 (62.3)	69 (60.5)	73 (64.0)	
Atrial fibrillation	79 (34.6)	42 (36.8)	37 (32.5)	
Atrial flutter	7 (3.1)	3 (2.6)	4 (3.5)	
Additional CTI ablation (%)	26 (11.4)	12 (10.5)	14 (12.3)	0.677
Duration of ablation [min]	70.0 (54.0, 90.0)	70.0 (53.0, 85.0)	70.0 (55.0, 90.0)	0.238
Fluoroscopy time [min]	10.4 (8.3, 14.5)	10.0 (8.2, 14.2)	11.0 (8.4, 14.6)	0.414
Contrast medium [ml]	18.2 ± 5.9	18.0 ± 5.0	18.4 ± 6.7	0.605
Electrical cardioversion (%)	95 (41.7)	46 (40.4)	49 (43.0)	0.687
Adverse events				
Stroke (%)	0 (0.0)	0 (0.0)	0 (0.0)	
TIA (%)	0 (0.0)	0 (0.0)	0 (0.0)	
Pericardial effusion (%)	0 (0.0)	0 (0.0)	0 (0.0)	
Cardiac tamponade (%)	0 (0.0)	0 (0.0)	0 (0.0)	
Myocardial infarction (%)	0 (0.0)	0 (0.0)	0 (0.0)	
Blood transfusion (%)	0 (0.0)	0 (0.0)	0 (0.0)	
Atrioesophageal fistula (%)	0 (0.0)	0 (0.0)	0 (0.0)	
Death (%)	0 (0.0)	0 (0.0)	0 (0.0)	
Puncture-site bleeding (%)	0 (0.0)	0 (0.0)	0 (0.0)	
Pseudoaneurysm (%)	0 (0.0)	0 (0.0)	0 (0.0)	
Arteriovenous fistula (%)	0 (0.0)	0 (0.0)	0 (0.0)	
Hematoma (%)	6 (2.6)	4 (3.5)	2 (1.8)	0.683
Pulmonary vein stenosis (%)	0 (0.0)	0 (0.0)	0 (0.0)	
Unresolved phrenic nerve injury at discharge (%)	5 (2.2)	2 (1.8)	3 (2.6)	1.000

Values are *n* (%), mean ± standard deviation, or median (25th–75th percentile)

*CTI*, cavotricuspid isthmus; *TIA*, transient ischemic attack

### Acute ablation success

In terms of acute ablation success, both CBs demonstrated high rates of final PV isolation with no statistical differences. The combined success rate for left superior PV (LSPV) and right superior PV (RSPV) isolation was 99.6%, while it was 100% for left inferior PV (LIPV) and 99.1% for right inferior PV (RIPV) (Table 3 and Supplementary Table S4). The rate of first-pass isolation, defined as successful isolation with a single cryoapplication (FAVI), did not differ between AFA-Pro and POLARx with respect to all PVs—exemplified by a FAVI rate of 90.4% and 91.2%, respectively (*p* = 0.984).

**Table 3:** Freezing characteristics of left superior pulmonary vein (LSPV)

	All patients (*n* = 228)	AFA-Pro (*n* = 114)	POLARx (*n* = 114)	*P* value
LSPV				
Rate of TTI recordings (%)	112 (49.3)	53 (46.9)	59 (51.8)	0.465
TTI [s]	48.0 (36.3, 61.8)	50.0 (36.0, 70.0)	42.0 (36.0, 58.0)	0.327
Duration of first freeze [s]	236.0 ± 44.4	244.1 ± 54.7	227.9 ± 29.1	0.006*
Total freeze time [s]	274.1 ± 126.1	271.1 ± 96.5	277.1 ± 150.1	0.719
Number of freezes [*n*]	1.0 (1.0, 1.0)	1.0 (1.0, 1.0)	1.0 (1.0, 1.0)	0.126
FAVI (%)	207 (91.2)	103 (90.4)	104 (91.2)	0.984
Final isolation of PV (%)	227 (99.6)	114 (100.0)	113 (99.1)	1.000
Minimal temperature [°C]	− 54.0 (− 60.0, − 48.0)	− 49.0 (− 53.0, − 46.0)	− 59.5 (− 62.0, − 55.0)	< 0.001*
Temperature at TTI [°C]	− 44.0 (− 49.0, − 39.0)	− 39.0 (− 42.5, − 35.0)	− 48.0 (− 50.0, − 44.0)	< 0.001*
Minimal esophagus temperature [°C]	34.0 (30.0, 35.4)	34.3 (30.2, 35.4)	33.4 (29.8, 35.3)	0.666
Temp. at 40 s [°C]	− 41.0 (− 47.0, − 35.0)	− 36.0 (− 38.0, − 32.0)	− 47.0 (− 49.0, − 43.0)	< 0.001*
Temp. at 60 s [°C]	− 46.0 (− 51.0, − 40.0)	− 41.0 (− 44.0, − 37.0)	− 51.0 (− 54.0, − 47.0)	< 0.001*
Time to − 30 °C [s]	30.0 (28.0, 33.0)	32.0 (29.5, 35.0)	29.0 (27.0, 31.0)	< 0.001*
Time to − 40 °C [s]	38.0 (32.0, 55.0)	55.0 (43.0, 74.0)	33.0 (31.0, 36.0)	< 0.001*
Thawing time to 0 °C [s]	15.0 (11.0, 22.0)	11.0 (8.0, 14.0)	21.0 (17.0, 24.0)	< 0.001*
Thawing time to 10 °C [s]	36.0 (28.0, 43.0)	35.0 (24.0, 43.0)	37.0 (30.0, 42.0)	0.184
AUC below 0 °C	11,203.0 (9900.5, 12,304.5)	9877.0 (9316.5, 10,756.5)	11,990.5 (11,316.8, 12,787.5)	< 0.001*
Mean temp. change between 20 and 40 s [°C/s]	− 1.5 ± 0.4	− 1.2 ± 0.2	− 1.8 ± 0.3	< 0.001*

Values are mean ± standard deviation or median (25th–75th percentile)

*TTI*, time to isolation; *FAVI*, first-pass isolation, first-attempt vein isolated; *PV*, pulmonary vein; *AUC*, area under the curve

The time to isolation (TTI) recordings for LSPV were available for 46.9% of patients treated with AFA-Pro and 51.8% of patients treated with POLARx, with no significant difference observed between the two CBs (*p* = 0.465) (Table 3). Similarly, there were no significant differences in TTI values between AFA-Pro and POLARx for LSPV, with median times of 50 s (36, 70) and 42 s (36, 58), respectively (*p* = 0.327). Analogous to these results, comparable rates and values for TTI were obtained for LIPV, RSPV, and RIPV (Supplementary Table S3).

### Freezing characteristics

**Fig. 1:**
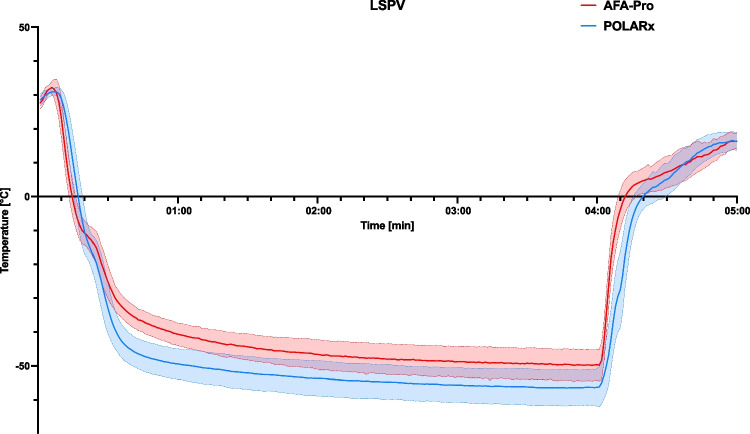
Time–temperature graph for cryoablation in the left superior pulmonary vein (LSPV) using AFA-Pro (*n* = 65 patients) and POLARx (*n* = 92 patients). Only patients with an ablation cycle duration of 240 s were included. The solid line corresponds to the mean, and the dashed line to the standard deviation

For LSPV, freezing characteristics are shown in Table 3 and Fig. 1.

For LIPV, RSPV, and RIPV, see Supplementary Table S4 and Supplementary Fig. S1.

With an average duration of 244.1 ± 54.7 s for AFA-Pro, the first ablation cycle of the LSPV was significantly longer compared to the POLARx CB with a duration of 227.9 ± 29.1 s (*p* = 0.006). This effect was also observed for LIPV. However, the total freeze time was comparable between both CBs in all PVs, including the LSPV (AFA-Pro: 271.1 ± 96.5 s vs. POLARx: 277.1 ± 150.1 s, *p* = 0.719). Additionally, there was no significant difference in the number of total freezes for all PVs.

The cooling profile analysis revealed distinct differences between the two CBs in terms of temperature characteristics and their ability to achieve and sustain low temperatures. POLARx achieved lower minimal temperatures in all PVs compared to AFA-Pro (e.g., LSPV: − 59.5 °C (− 62, − 55) vs. − 49 °C (− 53, − 46), *p* < 0.001) and lower temperatures at TTI (e.g., LSPV: − 48 °C (− 50, − 44) vs. − 39 °C (− 42.5, − 35), *p* < 0.001). Furthermore, the POLARx CB exhibited significantly lower temperatures after 40 and 60 s, as well as a larger mean temperature change between 20 and 40 s (e.g., LSPV: AFA-Pro − 1.2 ± 0.2 °C/s vs. POLARx − 1.8 ± 0.3 °C/s, *p* < 0.001). Consistent with these findings, POLARx reached low temperatures, such as − 40 °C, faster than AFA-Pro. The cooling performance of POLARx was further underscored by a significantly greater area under the curve (AUC) below 0 °C. Correspondingly, the thawing phase to reach 0 °C was significantly longer with POLARx compared to AFA-Pro (LSPV: AFA-Pro: 11.0 (8.0, 14.0) s vs. POLARx: 21.0 (17.0, 24.0) s, *p* < 0.001). With regards to safety, the minimal esophagus temperature in all PVs did not differ significantly between the two systems (e.g., LSPV: AFA-Pro: 34.25 °C (30.15, 35.40) vs. POLARx: 33.40 °C (29.80, 35.30), *p* = 0.666).

### Troponin levels in left atrium

The troponin levels in the left atrium were evaluated in an additional study cohort to assess myocardial injury during the ablation procedure and to compare the impact of both CB systems. Analysis indicated no significant differences in baseline parameters within the troponin cohort between the AFA-Pro and POLARx groups, as detailed in Supplementary Table S5. Additionally, procedural characteristics and the incidence of adverse events were comparable between the groups (Supplementary Table S6). Results concerning freezing characteristics were consistent with those observed in the main cohort: both catheters achieved a high rate of final isolation and the POLARx system demonstrated lower minimal temperatures and achieved a greater AUC except for the RSPV (*p* = 0.070 for nadir temperature, Supplementary Table S6).

**Fig. 2:**
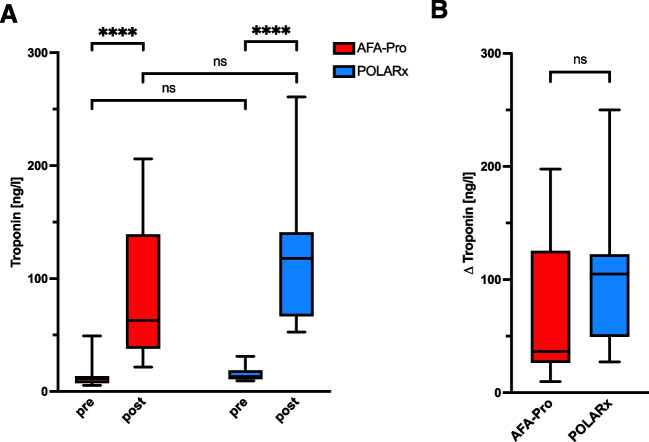
High-sensitive troponin levels in the left atrium for quantification of myocardial injury during the ablation procedure with AFA-Pro (*n* = 14) and POLARx (*n* = 16). **A** Comparison of pre- and post-procedural troponin release between the two cryoballoons. **B** Changes of pre- to post-procedural troponin (Δ Troponin) for AFA-Pro and POLARx. ****p < 0.0001, Mann–Whitney test

As expected, pre-ablation troponin levels were found to be low, with median levels of 11.6 (7.6, 13.5) ng/L for the AFA-Pro group and 13.2 (11.0, 18.8) ng/L for the POLARx group (*p* = 0.059) (Fig. 2A). Following PVI, both CBs led to a significant elevation in troponin levels within the left atrium: for AFA-Pro, the post-ablation median troponin level was 63.1 (38.1, 139.3) ng/L (*p* < 0.001), while POLARx resulted in a median troponin level of 118.0 (66.6, 141.0) ng/L (*p* < 0.001). When comparing the troponin levels after ablation between the two cryoballoons (Fig. 2A), higher numerical troponin values were observed with POLARx, although this difference did not reach statistical significance (*p* = 0.085). Analogously, POLARx also demonstrated a numerically higher troponin increase (AFA-Pro 36.3 (26.4, 125.4) ng/L vs. POLARx 104.9 (49.5, 122.2) ng/L), *p* = 0.077) (Fig. 2B). No correlation between troponin levels and the AUC was observed (data not shown).

### Adverse events

Table 2 gives detailed information about procedure-related complications. The intraprocedural analysis revealed a low incidence of adverse events with both AFA-Pro and POLARx CBs. Notably, no cases of neurological impairments, such as stroke or transient ischemic attacks (TIAs), were observed. Furthermore, none of the patients developed pericardial effusion or cardiac tamponade. Regarding groin site complications, there were no instances of relevant bleeding, pseudoaneurysm, or arteriovenous fistula. However, a total of six patients (2.6%) developed hematoma, with no statistically significant difference between the AFA-Pro and POLARx groups. A phrenic nerve palsy occurred in 5 patients (2.2%), with two patients (1.8%) in the AFA-Pro group and 3 patients (2.6%) in the POLARx group (*p* = 1.000).

## Discussion

In the first part of our study, we analyzed freezing characteristics of two different CB ablation systems (AFA-Pro and POLARx) for the treatment of AF and their possible implications on performance, acute ablation success, and safety, utilizing a propensity score-matched patient cohort. In a second part, we provide prospectively evaluated troponin measurements from the left atrium as quantifiable marker of myocardial tissue damage during the ablation procedure.

One of the notable observations from this study are the distinct differences in the freezing characteristics between the AFA-Pro and POLARx CBs. In line with previous studies [9, 11–15], POLARx achieved significant lower minimal temperatures in all PVs and exhibited a more rapid cooling rate compared to AFA-Pro, as evidenced by a more substantial mean temperature decrease observed within the 20 to 40-s interval. A greater AUC below 0 °C might also indicate a superior ability to sustain sub-zero temperatures. However, the pivotal question remains whether these improved freezing properties have practical meaningful implications on CB selection for PVI.

To date, several predictors regarding freezing characteristics have been identified for durable and efficient lesion formation during cryoablation. Nadir temperatures of − 53.5 °C [13] or even − 56 °C [16] for POLARx are independent predictors of acute and sustained (after a waiting period and adenosine testing) PVI. While thawing times of > 10 s to 0 °C were acceptable for the AFA [17], times of up to > 17 s are desirable for POLARx according to Iacopino and colleagues [16]. Accordingly, the minimum temperatures and thawing times measured in our work should indicate promising and effective isolation. Furthermore, an early attainment (< 60 s) of a temperature of at least − 40 °C predicts durable lesions during cryoablation with AFA [18, 19]. Although in our analysis both CBs reached − 40 °C in less than 1 min, the POLARx catheter reached this temperature significantly faster (median 31–33 s depending on PV). The need for adaptation of previously established predictors to accommodate the lower nadir temperatures and cooling profile of the POLARx CB suggests that the effective range of the catheter’s action spectrum has essentially been reduced to lower temperature levels.

The procedural characteristics presented, including the duration of the ablation procedure, amount of contrast medium used, and fluoroscopy time, were similar between the two CBs, indicating comparable procedural complexity and efficiency, but also feasibility. Although some studies reported longer procedure and fluoroscopy times, and higher contrast agent consumption with POLARx [9, 20], these differences may be attributed to a learning curve. Recent evidence suggests that as more experience is gained with POLARx, reports of comparable procedure characteristics to AFA-Pro are increasingly emerging [10, 11, 14].

Our results demonstrate that both AFA-Pro and POLARx achieved high rates of acute ablation success, with excellent isolation of PVs. The overall success rates for PVI, as well as the rates of first-pass isolations, were comparable between the two CB systems, indicating similar effectiveness in achieving the primary endpoint of AF ablation therapy. These findings are consistent with previous studies that have demonstrated the high acute success rates of AFA-Pro [7, 21, 22] and POLARx [8–10, 20, 23], but further studies are required to evaluate the long-term efficacy of POLARx.

A TTI < 60 s represents an additional powerful predictor of durable PVI [17, 19]. Our study did not identify any significant variations in the rate of TTI recordings or the actual TTI values between both CBs for all PVs. Of note, the temperatures reached at isolation were significantly lower with POLARx; however, the unaltered TTI suggests that these differences may not be clinically relevant.

The observed differences in freezing characteristics of POLARx, as compared to AFA-Pro, have become a focal point in contemporary electrophysiological research. Notably, several comparative studies [10, 11, 13–15, 23] have reported no significant discrepancies in key parameters such as minimal esophageal temperature, TTI, and procedural complications, specifically concerning phrenic nerve palsy. These findings have intensified the debate regarding whether the distinct freezing characteristics of POLARx are an actual physiological phenomenon or an artifact of measurement. Initial hypotheses proposed various explanations for these observations. Moser et al. [20] highlighted potential factors such as the lower internal balloon pressure of POLARx, differences in balloon expansion, and the more flexible design of the POLARSHEATH. Concurrently, Creta et al. [10] postulated that the unique material properties of POLARx might facilitate the formation of more antral oriented lesions. Significant insights were provided by Knecht et al., who dissected both ablation catheters and revealed noteworthy differences. Their findings included an altered positioning and injection orientation of the nitrous oxide injection coil in the POLARx, coupled with a reduced distance between the thermocouple and the gas outflow. Additionally, the nitrous oxide flow rate during the freezing process was higher in POLARx (7800 sccm) compared to AFA-Pro (7200 sccm), as detailed in the study by Guckel et al. [24]. Further elucidating this topic, Hayashi et al.’s work [25] in a porcine model provided pivotal data: by directly measuring myocardial tissue temperature, they demonstrated significantly lower temperatures during cryoablation with POLARx (− 58.4 °C ± 5.9 °C) as opposed to AFA-Pro (− 41.5 °C ± 4.9 °C, *p* < 0.001). This finding in a biological setting offers a crucial perspective on whether the technical distinctions between the two catheters translate to actual differences in tissue temperature during ablation.

In terms of safety, both CB systems demonstrated a high level of procedural safety with low complication rates that are in line with previous studies [9, 12, 23]. Although POLARx developed significant lower nadir temperatures, our analysis revealed no difference in minimal esophagus temperature or rate of phrenic nerve injuries. In our study, use of the DMS in combination with the POLARx CB did not reduce phrenic nerve palsy, and thus, there is currently no evidence in the literature supporting the clear benefit of the DMS. Further investigations are required to assess its potential advantages.

In recent years, the field of electrophysiology has witnessed substantial progress, particularly in the realms of ablation techniques and energy sources. Despite these advancements, a significant gap persists in our ability to directly evaluate the efficacy, durability, and overall success of lesions created during PVI. In this context, troponin, a biomarker typically released following cardiomyocyte injury, emerges as a potential candidate for assessing the acute impact of ablation procedures and quantifying thermal tissue damage. Previous investigations have primarily focused on troponin measurements following radiofrequency (RF) ablation, examining correlations with cumulative RF energy, duration of energy application, lesion size, and lesion type (linear vs. focal) [26–28]. Subsequent studies have compared troponin release across different energy sources, notably RF and CB ablation, with markedly diverse findings. Some researchers reported higher troponin levels post-RF ablation [29, 30], while others observed elevated levels following CB ablation [31] or found no significant differences [32]. These studies’ comparability is hindered by variations in the ablation techniques employed, the specific troponin assays used, and the timing of blood sample collection. Our study uniquely compares troponin levels between two catheters employing the same ablation technique in patients with AF. We aimed to quantify and compare myocardial injury following AFA-Pro and POLARx ablation. We observed median troponin levels of 63 ng/L for AFA-Pro and 118 ng/L for POLARx. Prior studies reported varying high-sensitive troponin levels: 850 ng/L after 4 h [33], 806–840 ng/L after 18–24 h [32], and up to approximately 1500 ng/L after 1 day [34]. The lower troponin values in our study could be attributed to the immediate post-ablation blood sample collection, aligning with the short-term collection reported by other authors (151 pg/mL by Lermoine et al. [35], 168 pg/mL by Scherschel et al. [36]). Furthermore, our study’s unique approach of drawing blood directly from the cryoballoon sheath within the left atrium contrasts with the peripheral blood sampling in previous studies, possibly contributing to the observed troponin value differences. Interestingly, we noted a trend towards higher post-ablation troponin levels in the POLARx group, along with a numerically higher troponin delta. These results might indicate a greater degree of myocardial injury attributed to the altered freezing characteristics of the POLARx CB, and statistical significance might have been missed due to the low number of patients. Despite these observations, no impact on clinical endpoints, such as feasibility, performance, efficacy, and safety, was apparent. The relationship between troponin release, long-term outcomes, and procedural safety remains a subject of debate and warrants further investigation. In our study, no increase in procedural complications was observed in patients with elevated troponin levels, although the small patient population for troponin determinations limited the study’s power in this aspect.

## Limitations

While our study provides valuable insights into the comparison of AFA-Pro and POLARx for AF ablation, there are certain limitations that should be considered. First, this was a retrospective, observational study conducted at a single center, which may limit the generalizability of the findings. Second, the study focused on intraprocedural analyses and acute ablation outcomes. Long-term follow-up data, including recurrence rates of AF and clinical outcomes, were not included in this analysis. Future studies should investigate the long-term efficacy and safety of AFA-Pro and POLARx in larger patient cohorts. Furthermore, the sample size for troponin analysis was relatively small, which may have limited the statistical power to detect differences.

## Conclusion

In this study, we identified significant differences in the freezing characteristics of two cryoballoon ablation systems, AFA-Pro and POLARx. Yet these differences did not lead to significant variations in clinical performance, ablation success, or safety. Despite POLARx’s lower temperatures potentially causing greater myocardial injury as evidenced by numerically higher troponin levels, no impact on essential clinical outcomes was observed. Further research is needed to fully comprehend the clinical implications of these differences and to confirm the long-term effectiveness and safety of the POLARx system.

## Supplementary Information

Below is the link to the electronic supplementary material.

Supplementary file1 (EPS 2381 KB)

Supplementary file2 (DOC 59.5 KB)

Supplementary file3 (DOC 52 KB)

Supplementary file4 (DOC 55 KB)

Supplementary file5 (DOC 148 KB)

Supplementary file6 (DOC 68 KB)

Supplementary file7 (DOC 93.5 KB)

## Data Availability

The data that support the findings of this study are available from the corresponding author upon reasonable request.
