# Supplementary material for: Comparison of Arctic Front Advance Pro and POLARx cryoballoons for ablation therapy of atrial fibrillation: an intraprocedural analysis
Source: Clin Res Cardiol. 2024 Feb 15;114(1):83–92. doi: 10.1007/s00392-024-02398-2 (PMC11772469; doi:10.1007/s00392-024-02398-2)
Supplement: Supplementary file 2 — Supplementary file2 (DOC 59.5 KB) [file 392_2024_2398_MOESM2_ESM.doc]

**Supplementary Table 1.** Medication of study population.

|  | **All patients (n = 228)** | **AFA-Pro (n = 114)** | **POLARx (n = 114)** | **P value** |
| --- | --- | --- | --- | --- |
|  |  |  |  |  |
| ACE inhibitor (%) | 57 (25.0) | 31 (27.2) | 26 (22.8) | 0.444 |
| AT-1 blocker (%) | 91 (39.9) | 41 (36.0) | 51 (43.9) | 0.224 |
| Calcium channel blockers (%) | 2 (0.9) | 0 (0.0) | 2 (1.8) | 0.498 |
| Digoxin/digitoxin (%) | 4 (1.8) | 1 (0.9) | 3 (2.6) | 0.622 |
| Diuretic (%) | 95 (41.7) | 48 (42.1) | 47 (41.2) | 0.893 |
| Amiodarone (%) | 23 (10.1) | 12 (10.5) | 11 (9.6) | 0.826 |
| Dronedaron (%) | 1 (0.4) | 0 (0.0) | 1 (0.9) | 1.000 |
| Class 1C antiarrhythmic (%) | 26 (11.4) | 14 (12.3) | 12 (10.5) | 0.677 |
| Beta-blocker (%) | 194 (85.1) | 102 (89.5) | 92 (80.7) | 0.063 |
| Sotalol (%) | 1 (0.4) | 0 (0.0) | 1 (0.9) | 1.000 |
| Clopidogrel (%) | 10 (4.4) | 2 (1.8) | 8 (7.0) | 0.052 |
| Phenprocoumon (%) | 4 (1.8) | 1 (0.9) | 3 (2.6) | 0.622 |
| Direct oral anticoagulant (%) | 224 (98.2) | 113 (99.1) | 111 (97.4) | 0.622 |
| Dabigatran (%) | 28 (12.3) | 12 (10.5) | 16 (14.0) | 0.420 |
| Rivaroxaban (%) | 46 (20.2) | 23 (20.2) | 23 (20.2) | 1.000 |
| Apixaban (%) | 104 (45.6) | 57 (50.0) | 47 (41.2) | 0.184 |
| Edoxaban (%) | 46 (20.2) | 21 (18.4) | 25 (21.9) | 0.509 |
|  |  |  |  |  |

Values are n (%), mean ± standard deviation or median (25^th^–75^th^ percentile). Calcium channel blockers: only verapamil and diltiazem included.
