# Supplementary material for: Comparison of Arctic Front Advance Pro and POLARx cryoballoons for ablation therapy of atrial fibrillation: an intraprocedural analysis
Source: Clin Res Cardiol. 2024 Feb 15;114(1):83–92. doi: 10.1007/s00392-024-02398-2 (PMC11772469; doi:10.1007/s00392-024-02398-2)
Supplement: Supplementary file 3 — Supplementary file3 (DOC 52 KB) [file 392_2024_2398_MOESM3_ESM.doc]

**Supplementary Table 2.** Laboratory parameters of study population.

|  | | **All patients (n = 228)** | **AFA-Pro (n = 114)** | | **POLARx (n = 114)** | | **P value** |
| --- | --- | --- | --- | --- | --- | --- | --- |
|  | |  |  | |  | |  |
| Creatinine [mg/dl] | | 0.9 (0.8, 1.1) | 0.9 (0.8, 1.1) | | 0.9 (0.8, 1.1) | | 0.319 |
| INR | | 1.1 (1.0, 1.2) | 1.1 (1.0, 1.2) | | 1.1 (1.0, 1.2) | | 0.204 |
| TSH [µU/ml] | | 1.4 (1.0, 2.0) | 1.4 (0.9, 2.0) | | 1.5 (1.0, 2.0) | | 0.610 |
| Hemoglobin [g/dl] | | 14.0 (13.1, 15.0) | 14.0 (12.8, 14.9) | | 13.9 (13.2, 15.0) | | 0.471 |
| Leucocytes [G/l] | | 7.1 (6.2, 8.2) | 6.9 (5.9, 8.1) | | 7.2 (6.4, 8.3) | | 0.235 |
| Neutrophils [G/l] | | 4.6 ± 1.4 | 4.5 ± 1.4 | | 4.7 ± 1.4 | | 0.623 |
| CRP [mg/l] | | 1.6 (0.9, 3.0) | 1.8 (0.9, 2.4) | | 1.3 (0.9, 3.1) | | 0.622 |
|  |  | | |  | |  |  |

Values are n (%), mean ± standard deviation or median (25^th^–75^th^ percentile).

INR: international normalized ratio. TSH: thyroid-stimulating hormone. CRP: c-reactive protein.
