# Supplementary material for: Comparison of Arctic Front Advance Pro and POLARx cryoballoons for ablation therapy of atrial fibrillation: an intraprocedural analysis
Source: Clin Res Cardiol. 2024 Feb 15;114(1):83–92. doi: 10.1007/s00392-024-02398-2 (PMC11772469; doi:10.1007/s00392-024-02398-2)
Supplement: Supplementary file 4 — Supplementary file4 (DOC 55 KB) [file 392_2024_2398_MOESM4_ESM.doc]

**Supplementary Table 3.** Echocardiographic characteristics of study population.

|  | **All patients (n = 228)** | **AFA-Pro (n = 114)** | **POLARx (n = 114)** | **P value** |
| --- | --- | --- | --- | --- |
|  |  |  |  |  |
| LVEF [%] | 58.0 (55.4, 63.5) | 57.8 (55.1, 63.6) | 58.1 (55.7, 63.5) | 0.723 |
| LA volume [ml] | 59.1 (45.7, 81.9) | 60.0 (45.8, 85.9) | 57.7 (43.7, 81.4) | 0.534 |
| LV volume [ml] | 103.0 (71.4, 128.9) | 103.8 (72.3, 126.2) | 100.8 (70.4, 131.5) | 0.693 |
| sPAP > 25 mmHg (%) | 73 (32.3) | 41 (36.3) | 32 (28.3) | 0.200 |
| MR > I° (%) | 30 (13.2) | 17 (14.9) | 13 (11.4) | 0.433 |
| LAA velocity < 0,4 m/s (%) | 25 (11.2) | 11 (9.8) | 14 (12.5) | 0.524 |
| PFO (%) | 24 (10.5) | 11 (9.6) | 13 (11.4) | 0.666 |
| ASD (%) | 4 (1.8) | 2 (1.8) | 2 (1.8) | 1.000 |

Values are n (%), mean ± standard deviation or median (25^th^–75^th^ percentile).

LVEF: left ventricular ejection fraction. LA volume: left atrial volume. LV volume: left ventricle volume. sPAP: systolic pulmonary artery pressure. MR: mitral regurgitation. LAA velocity: left atrial appendage velocity. PFO: patent foramen ovale. ASD: atrial septal defect.
