# Supplementary material for: Comparison of Arctic Front Advance Pro and POLARx cryoballoons for ablation therapy of atrial fibrillation: an intraprocedural analysis
Source: Clin Res Cardiol. 2024 Feb 15;114(1):83–92. doi: 10.1007/s00392-024-02398-2 (PMC11772469; doi:10.1007/s00392-024-02398-2)
Supplement: Supplementary file 5 — Supplementary file5 (DOC 148 KB) [file 392_2024_2398_MOESM5_ESM.doc]

**Supplementary Table 4.** Freezing characteristics of left inferior pulmonary vein (LIPV), right superior pulmonary vein (RSPV) and right inferior pulmonary vein (RIPV)

|  | **All patients (n = 228)** | **AFA-Pro (n = 114)** | **POLARx (n = 114)** | **P value** |
| --- | --- | --- | --- | --- |
|  |  |  |  |  |
| **LIPV** |  |  |  |  |
| Rate of TTI recordings (%) | 97 (42.7) | 42 (37.2) | 55 (48.2) | 0.092 |
| TTI [s] | 36.0 (27.0, 62.0) | 37.5 (26.5, 75.5) | 35.0 (27.0, 54.0) | 0.413 |
|  |  |  |  |  |
| Duration of first freeze [s] | 232.9 ± 53.7 | 246.4 ± 63.3 | 219.6 ± 38.0 | <0.001* |
| Total freeze time [s] | 270.2 ± 111.8 | 284.6 ± 128.8 | 256.0 ± 90.4 | 0.054 |
| Number of freezes [n] | 1.0 (1.0, 1.0) | 1.0 (1.0, 1.0) | 1.0 (1.0, 1.0) | 0.471 |
|  |  |  |  |  |
| FAVI (%) | 204 (89.9) | 102 (90.3) | 102 (89.5) | 0.843 |
| Final Isolation of PV (%) | 227 (100.0) | 114 (100.0) | 114 (100.0) |  |
|  |  |  |  |  |
| Minimal Temperature [°C] | -51.0 (-58.0, -46.0) | -46.0 (-51.0, -44.0) | -57.0 (-60.0, -53.0) | <0.001* |
| Temperature at TTI [°C] | -38.0 (-44.0, -26.0) | -36.0 (-38.0, -25.5) | -43.0 (-47.0, -31.0) | <0.001* |
| Minimal esophagus temperature [°C] | 32.0 (23.1, 35.0) | 33.4 (24.5, 35.0) | 31.9 (21.7, 35.0) | 0.438 |
|  |  |  |  |  |
| Temp. at 40 s [°C] | -39.0 (-45.0, -34.0) | -34.0 (-38.0, -31.0) | -45.0 (-48.0, -41.0) | <0.001* |
| Temp. at 60 s [°C] | -43.0 (-49.0, -38.0) | -39.0 (-42.0, -36.0) | -49.0 (-52.0, -45.8) | <0.001* |
|  |  |  |  |  |
| Time to -30 °C [s] | 28.0 (26.0, 32.0) | 31.0 (28.0, 36.0) | 27.0 (25.0, 29.0) | <0.001* |
| Time to -40 °C [s] | 39.0 (32.0, 59.0) | 58.5 (47.8, 93.5) | 32.0 (30.0, 36.0) | <0.001* |
|  |  |  |  |  |
| Thawing Time to 0 °C [s] | 13.0 (9.0, 19.0) | 9.0 (7.0, 11.0) | 19.0 (15.5, 22.5) | <0.001* |
| Thawing Time to 10 °C [s] | 30.0 (22.0, 37.0) | 27.0 (18.0, 34.0) | 32.0 (26.0, 38.0) | <0.001* |
|  |  |  |  |  |
| AUC below 0 °C | 10581.5 (9707.3, 11765.3) | 9715.5 (9213.3, 10087.3) | 11668.5 (10888.8, 12297.0) | <0.001* |
|  |  |  |  |  |
| Mean temp. change between 20 and 40 s [°C/s] | -1.2 ± 0.4 | -1.0 ± 0.4 | -1.5 ± 0.2 | <0.001* |

Values are mean ± standard deviation or median (25^th^–75^th^ percentile).

TTI: time to isolation. FAVI: first-pass isolation, first-attempt vein isolated. PV: pulmonary vein. AUC: area under the curve.

|  | **All patients (n = 228)** | **AFA-Pro (n = 114)** | **POLARx (n = 114)** | **P value** |
| --- | --- | --- | --- | --- |
|  |  |  |  |  |
| **RSPV** |  |  |  |  |
| Rate of TTI recordings (%) | 71 (31.3) | 39 (34.2) | 32 (28.1) | 0.317 |
| TTI [s] | 32.0 (28.0, 49.8) | 32.0 (28.0, 50.0) | 34.0 (27.5, 55.5) | 0.480 |
|  |  |  |  |  |
| Duration of first freeze [s] | 220.0 ± 46.8 | 217.8 ± 56.3 | 222.3 ± 35.0 | 0.464 |
| Total freeze time [s] | 261.9 ± 116.2 | 253.8 ± 117.9 | 269.9 ± 114.5 | 0.295 |
| Number of freezes [n] | 1.0 (1.0, 1.0) | 1.0 (1.0, 1.0) | 1.0 (1.0, 1.0) | 0.225 |
|  |  |  |  |  |
| FAVI (%) | 203 (89.0) | 104 (91.2) | 99 (86.8) | 0.289 |
| Final Isolation of PV (%) | 227 (99.6) | 114 (100) | 113 (99.1) | 1.000 |
|  |  |  |  |  |
| Minimal Temperature [°C] | -53.8 ± 7.4 | -50.3 ± 5.2 | -57.2 ± 7.7 | <0.001* |
| Temperature at TTI [°C] | -38.5 ± 10.8 | -34.2 ± 7.8 | -43.6 ± 11.7 | <0.001* |
| Minimal esophagus temperature [°C] | 35.2 (34.8, 35.5) | 35.2 (34.8, 35.5) | 35.0 (34.8, 35.5) | 0.857 |
|  |  |  |  |  |
| Temp. at 40 s [°C] | -41.0 (-47.0, -36.0) | -37.0 (-41.0, -33.0) | -47.0 (-50.0, -43.0) | <0.001* |
| Temp. at 60 s [°C] | -46.0 (-51.0, -41.0) | -42.0 (-45.3, -38.0) | -50.0 (-55.0, -47.0) | <0.001* |
|  |  |  |  |  |
| Time to -30 °C [s] | 27.0 (25.0, 30.8) | 29.0 (27.0, 33.0) | 26.0 (24.0, 28.0) | <0.001* |
| Time to -40 °C [s] | 35.5 (31.0, 50.0) | 48.0 (37.0, 67.0) | 31.0 (28.0, 34.0) | <0.001* |
|  |  |  |  |  |
| Thawing Time to 0 °C [s] | 14.0 (10.0, 19.0) | 10.0 (8.0, 13.0) | 19.0 (15.0, 24.0) | <0.001* |
| Thawing Time to 10 °C [s] | 32.0 (24.0, 41.0) | 29.0 (19.0, 41.0) | 32.0 (26.0, 42.0) | 0.027* |
|  |  |  |  |  |
| AUC below 0 °C | 10952.5 (9918.3, 12006.5) | 9905.0 (9526.0, 10412.0) | 11825.0 (10931.0, 12611.0) | <0.001* |
|  |  |  |  |  |
| Mean temp. change between 20 and 40 s [°C/s] | -1.3 ± 0.3 | -1.1 ± 0.2 | -1.5 ± 0.2 | <0.001* |

Values are mean ± standard deviation or median (25^th^–75^th^ percentile).

TTI: time to isolation. FAVI: first-pass isolation, first-attempt vein isolated. PV: pulmonary vein. AUC: area under the curve.

|  | **All patients (n = 228)** | **AFA-Pro (n = 114)** | **POLARx (n = 114)** | **P value** |
| --- | --- | --- | --- | --- |
|  |  |  |  |  |
| **RIPV** |  |  |  |  |
| Rate of TTI recordings (%) | 79 (35.0) | 43 (38.1) | 36 (31.9) | 0.329 |
| TTI [s] | 45.0 (33.0, 62.0) | 42.0 (33.0, 62.0) | 50.0 (32.0, 65.3) | 0.494 |
|  |  |  |  |  |
| Duration of first freeze [s] | 221.85 ± 55.6 | 223.2 ± 62.4 | 220.5 ± 48.1 | 0.708 |
| Total freeze time [s] | 259.6 ± 109.0 | 254.3 ± 103.9 | 265.0 ± 114.1 | 0.465 |
| Number of freezes [n] | 1.0 (1.0, 1.0) | 1.0 (1.0, 1.0) | 1.0 (1.0, 1.0) | 0.640 |
|  |  |  |  |  |
| FAVI (%) | 195 (87.1) | 102 (90.3) | 93 (83.8) | 0.149 |
| Final Isolation of PV (%) | 225 (99.1) | 114 (100) | 111 (98.2) | 0.247 |
|  |  |  |  |  |
| Minimal Temperature [°C] | -52.0 (-58.0, -47.0) | -48.0 (-52.0, -45.0) | -56.0 (-61.0, -52.0) | <0.001* |
| Temperature at TTI [°C] | -41.0 (-47.0, -36.0) | -38.0 (-41.0, -34.0) | -47.0 (-51.0, -40.3) | <0.001* |
| Minimal esophagus temperature [°C] | 35.0 (31.4, 35.3) | 34.7 (26.1, 35.3) | 35.0 (32.6, 35.3) | 0.378 |
|  |  |  |  |  |
| Temp. at 40 s [°C] | -40.0 (-44.0, -36.0) | -36.0 (-38.0, -33.0) | -44.0 (-47.0, -41.0) | <0.001* |
| Temp. at 60 s [°C] | -43.0 (-48.0, -39.0) | -40.0 (-43.0, -37.0) | -48.0 (-51.3, -44.0) | <0.001* |
|  |  |  |  |  |
| Time to -30 °C [s] | 28.0 (26.0, 31.0) | 30.0 (27.0, 32.5) | 27.0 (25.0, 29.0) | <0.001* |
| Time to -40 °C [s] | 39.0 (32.0, 56.0) | 52.0 (42.0, 73.0) | 33.0 (29.8, 38.3) | <0.001* |
|  |  |  |  |  |
| Thawing Time to 0 °C [s] | 13.5 (9.0, 20.0) | 9.0 (7.0, 12.0) | 20.0 (16.0, 25.5) | <0.001* |
| Thawing Time to 10 °C [s] | 29.0 (22.0, 40.0) | 26.0 (14.0, 35.5) | 34.0 (27.0, 42.5) | <0.001* |
|  |  |  |  |  |
| AUC below 0 °C | 10876.5 (9967.8, 12085.8) | 9994.0 (9435.0, 10449.5) | 11624.0 (10683.0, 12658.0) | <0.001* |
|  |  |  |  |  |
| Mean temp. change between 20 and 40 s [°C/s] | -1.2 ± 0.3 | -1.0 ± 0.2 | -1.4 ± 0.4 | <0.001* |

Values are mean ± standard deviation or median (25^th^–75^th^ percentile).

TTI: time to isolation. FAVI: first-pass isolation, first-attempt vein isolated. PV: pulmonary vein. AUC: area under the curve.
