# Supplementary material for: Comparison of Arctic Front Advance Pro and POLARx cryoballoons for ablation therapy of atrial fibrillation: an intraprocedural analysis
Source: Clin Res Cardiol. 2024 Feb 15;114(1):83–92. doi: 10.1007/s00392-024-02398-2 (PMC11772469; doi:10.1007/s00392-024-02398-2)
Supplement: Supplementary file 6 — Supplementary file6 (DOC 68 KB) [file 392_2024_2398_MOESM6_ESM.doc]

**Supplementary Table 5.** Baseline characteristics of troponin cohort.

|  | **All patients (n = 30)** | **AFA-Pro (n = 14)** | **POLARx (n = 16)** | **P value** |
| --- | --- | --- | --- | --- |
|  |  |  |  |  |
| Age [years] | 66.4 ± 10.4 | 65.3 ± 9.3 | 67.3 ± 11.5 | 0.604 |
| Male sex (%) | 24 (80.0) | 13 (92.9) | 11 (68.8) | 0.175 |
| Persistent AF (%) | 13 (43.3) | 7 (50.0) | 6 (37.5) | 0.491 |
| CHA_2_DS_2_-VASc score | 2.1 ± 1.5 | 2.0 ± 1.4 | 2.1 ± 1.7 | 0.825 |
| HASBLED score | 2.2 ± 0.9 | 2.2 ± 1.0 | 2.2 ± 0.9 | 0.939 |
|  |  |  |  |  |
| Hypertension (%) | 19 (63.3) | 10 (71.4) | 9 (56.3) | 0.389 |
| Dyslipidemia (%) | 9 (30.0) | 6 (42.9) | 3 (18.8) | 0.236 |
| BMI [kg/m^2^] | 27.6 ± 4.3 | 28.4 ± 5.4 | 28.8 ± 2.9 | 0.310 |
| Obesity (%) | 9 (30.0) | 6 (42.9) | 3 (18.8) | 0.236 |
| Diabetes (%) | 2 (6.7) | 0 (0.0) | 2 (12.5) | 0.485 |
| Coronary artery disease (%) | 7 (23.3) | 4 (28.6) | 3 (18.8) | 0.675 |
| Congestive heart failure (%) | 4 (13.3) | 3 (21.4) | 1 (6.3) | 0.315 |
| Peripheral artery disease (%) | 1 (3.3) | 0 (0.0) | 1 (6.3) | 1.000 |
| Carotid artery disease (%) | 1 (3.3) | 0 (0.0) | 1 (6.3) | 1.000 |
| Previous stroke (%) | 0 (0.0) | 0 (0.0) | 0 (0.0) |  |
| Previous TIA (%) | 1 (3.3) | 0 (0.0) | 1 (6.3) | 1.000 |
| COPD (%) | 1 (3.3) | 0 (0.0) | 1 (6.3) | 1.000 |
| Sleep apnea (%) | 1 (3.3) | 0 (0.0) | 1 (6.3) | 1.000 |

Values are n (%), mean ± standard deviation or median (25^th^–75^th^ percentile).

AF: atrial fibrillation. BMI: body mass index. Obesity was defined as BMI ≥ 30 kg/m². TIA: transient ischaemic attack. COPD: chronic obstructive pulmonary disease.
