# Supplementary material for: Comparison of Arctic Front Advance Pro and POLARx cryoballoons for ablation therapy of atrial fibrillation: an intraprocedural analysis
Source: Clin Res Cardiol. 2024 Feb 15;114(1):83–92. doi: 10.1007/s00392-024-02398-2 (PMC11772469; doi:10.1007/s00392-024-02398-2)
Supplement: Supplementary file 7 — Supplementary file7 (DOC 93.5 KB) [file 392_2024_2398_MOESM7_ESM.doc]

**Supplementary Table 6.** Procedural characteristics, adverse events and freezing characteristics of troponin cohort.

|  | **All patients (n = 30)** | **AFA-Pro (n = 14)** | **POLARx (n = 16)** | **P value** |
| --- | --- | --- | --- | --- |
|  |  |  |  |  |
| Preprocedural rhythm (%)  Sinus rhythm  Atrial fibrillation  Atrial flutter | 15 (50.0)  12 (40.0)  3 (10.0) | 5 (35.7)  7 (50.0)  2 (14.3) | 10 (62.5)  5 (31.3)  1 (6.3) | 0.331 |
| Additional CTI ablation (%) | 4 (13.3) | 2 (14.3) | 2 (12.5) | 1.000 |
| Duration of ablation [min] | 76.0 (64.0, 85.5) | 73.0 (59.8, 83.5) | 76.0 (69.0, 89.0) | 0.112 |
| Fluoroscopy time [min] | 12.9 (9.3, 15.0) | 9.5 (7.4, 14.3) | 13.3 (9.4, 15.2) | 0.193 |
| Contrast medium [ml] | 18.0 ± 5.9 | 16.9 ± 4.7 | 18.4 ± 6.4 | 0.564 |
| Electrical cardioversion (%) | 16 (53.3) | 10 (71.4) | 6 (37.5) | 0.063 |
| **Adverse Events** |  |  |  |  |
| Stroke (%) | 0 (0.0) | 0 (0.0) | 0 (0.0) |  |
| TIA (%) | 0 (0.0) | 0 (0.0) | 0 (0.0) |  |
| Pericardial effusion (%) | 0 (0.0) | 0 (0.0) | 0 (0.0) |  |
| Cardiac tamponade (%) | 0 (0.0) | 0 (0.0) | 0 (0.0) |  |
| Myocardial infarction (%) | 0 (0.0) | 0 (0.0) | 0 (0.0) |  |
| Blood transfusion (%) | 0 (0.0) | 0 (0.0) | 0 (0.0) |  |
| Atrioesophageal fistula (%) | 0 (0.0) | 0 (0.0) | 0 (0.0) |  |
| Death (%) | 0 (0.0) | 0 (0.0) | 0 (0.0) |  |
| Puncture-site bleeding (%) | 1 (3.3) | 0 (0.0) | 1 (6.3) | 1.000 |
| Pseudoaneurysm (%) | 0 (0.0) | 0 (0.0) | 0 (0.0) |  |
| Arteriovenous fistula (%) | 0 (0.0) | 0 (0.0) | 0 (0.0) |  |
| Hematoma (%) | 1 (3.3) | 1 (7.1) | 0 (0.0) | 0.467 |
| Pulmonary vein stenosis (%) | 0 (0.0) | 0 (0.0) | 0 (0.0) |  |
| Unresolved phrenic nerve injury at discharge (%) | 0 (0.0) | 0 (0.0) | 0 (0.0) |  |
|  |  |  |  |  |
| **LSPV** |  |  |  |  |
| Final Isolation of PV (%) | 30 (100.0) | 14 (100.0) | 16 (100.0) |  |
| Minimal Temperature [°C] | -55.0 (-59.3, -51.0) | -51.5 (-55.0, -46.0) | -58.5 (-64.0, -52.3) | 0.004* |
| AUC below 0 °C | 11289.0 (10396.5, 12158.3) | 11000.5 (10123.3, 11534.3) | 11769.0 (10376.8, 12331.3) | 0.027* |
| **LIPV** |  |  |  |  |
| Final Isolation of PV (%) | 30 (100.0) | 14 (100.0) | 16 (100.0) |  |
| Minimal Temperature [°C] | -50.5 (-55.3, -47.0) | -47.0 (-51.3, -45.8) | -53.5 (-57.8, -50.3) | 0.009* |
| AUC below 0 °C | 10437.5 (9986.3, 12051.3) | 10000.5 (9477.5, 10259.8) | 11395.5 (10455.3, 12312.0) | 0.006* |
| **RSPV** |  |  |  |  |
| Final Isolation of PV (%) | 30 (100.0) | 14 (100.0) | 16 (100.0) |  |
| Minimal Temperature [°C] | -57.0 (-60.3, -53.0) | -55.0 (-60.0, -49.8) | -57.5 (-63.8, -54.3) | 0.070 |
| AUC below 0 °C | 11756.0 (10478.5, 12502.5) | 11118.0 (9465.0, 12712.5) | 11845.0 (11093.3, 12453.5) | 0.106 |
| **RIPV** |  |  |  |  |
| Final Isolation of PV (%) | 30 (100.0) | 14 (100.0) | 16 (100.0) |  |
| Minimal Temperature [°C] | -55.5 (-59.3, -48.8) | -50.0 (-55.3, -45.5) | -59.0 (-61.8, -55.3) | <0.001* |
| AUC below 0 °C | 11452.0 (9687.0, 12090.5) | 10160.5 (8505.0, 10604.8) | 11937.0 (10985.0, 12485.3) | <0.001* |

Values are mean ± standard deviation or median (25^th^–75^th^ percentile).

CTI: cavotricuspid isthmus. TIA: transient ischaemic attack. PV: pulmonary vein. AUC: area under the curve.
